# Deaths among young people in England increased significantly in 10 of 11 weeks after COVID-19 vaccination and doubled in three

**DOI:** 10.17179/excli2024-7498

**Published:** 2024-07-04

**Authors:** Jarle Aarstad

**Affiliations:** 1 HVL Business School, Western Norway University of Applied Science, PO Box 7030, NO-5020 Bergen, Norway

## ⁯⁯⁯

Studying cohorts of young people in England aged 12-29, this research letter found that deaths increased significantly (95 % CIs) in 10 of 11 weeks after COVID-19 vaccination compared to the first week. In three of those weeks, deaths doubled. The pattern was similar for each dose, e.g., the incidence rate ratio (IRR) was 1.95 (95 % CI 1.15-3.39) and 2.58 (95 % CI 1.33-5.03) four weeks after doses one and two, and 2.67 (95 % CI 1.04-6.81) six weeks after dose three. The findings diverge from Nafilyan et al. (2023[9]), analyzing similar data from England, likely because they compared deaths during the first 12 weeks after vaccination with later deaths, labeled as a self-controlled case series method (Petersen et al., 2016[11]). Hence, their conclusion hinged on assuming a zero increased risk of dying more than 12 weeks after vaccination, but if the risk were increased, it could mask the findings I uncovered. Finally, Nafilyan et al. (2023[9]) found fewer deaths between the first and 24th week after vaccination than later, which disturbingly indicates increased long-term mortality (later than 24 weeks).

Since research on COVID-19 vaccination side effects has indicated that they occur shortly after injection (Faksova et al., 2024[4]), including among young (Karlstad et al., 2022[7]), comparing deaths in the first 12 weeks with deaths later as a reference period may be defendable. However, without any obvious explanation, many countries have experienced excess deaths after the COVID-19 pandemic's peak (Mostert et al., 2024[8]), and data among young show similar trends (Wilthil, 2024[16]). Consequently, as excess deaths are largely unexplained, along with randomized trials showing COVID-19 vaccinees with an increased risk of adverse events (Fraiman et al., 2022[5]), one should be cautious about using a time window later than 12 weeks after vaccination as a reference period. The cautiousness further concurs with the fact that “[i]n rare cases long-term side effects lasting more than three months may occur” (Norwegian Institute of Public Health, 2024[10]). Also, pre-COVID-19 data have shown a higher probability of dying from myocarditis, a known side effect (Karlstad et al., 2022[7]), later than before three months (Chang et al., 2017[3]). 

Taken together, not ruling out that using a time window later than 12 weeks after vaccination as a reference period may mask an increase in deaths earlier, I instead primarily compared deaths in the first week after vaccination with deaths in the following weeks. I.e., I used the first week after vaccination as a reference period. Empirically, I analyzed cohorts of young people in England, 12-29 years, during 12 weeks after their first, second, and third COVID-19 vaccination.

Side effects may have increased deaths even in the first week after vaccination, implying that my estimation approach is conservative. I.e., using the first week after vaccination as a reference period could eventually mask effects similar to those addressed above. I nonetheless assume a low increase of deaths in the first week after vaccination compared to assuming no vaccination as, despite side effects “usually occur during the first 1-2 days” (Norwegian Institute of Public Health, 2024[10]), there is always a time lag between symptoms and death. The assumption aligns with Nafilyan et al. (2023[9]), showing that the risk of dying one week after vaccination was significantly lower (95 % CIs) compared to the reference period after 12 weeks for each of the three first doses (Figure 1b in their study). 

The UK Office for National Statistics (Bermingham and Nafilyan, 2023[1], Table 5) provided data on deaths among young people in England, 12-29 years, in the 12 weeks following the COVID-19 vaccination, with doses one, two, and three. Also, they provided data on the number of people in England between 12 and 17 (Judd et al., 2022[6], Table 1) and 18 and 29 years (Willson et al., 2023[15], Table 1) receiving at least one, two, and three COVID-19 vaccination doses, and the age groups were summarized. (To estimate those between 18 and 29 receiving at least one dose, I summarized those receiving dose one only, dose two only, dose three only and at least four doses. I followed similar procedures for doses two and three.) I merged the data from the different sources. Those dying in the first week after dose one were subtracted from the vaccinated population when estimating week two, etc., and I followed a similar approach for doses two and three. 

Supplementary Figure 1A shows deaths per million each week since COVID-19 vaccination. It includes all doses combined and each individually. Deaths appear to be higher in the weeks following the first one, perhaps except for the last week or weeks of observation. Also, there appear to be fewer deaths after doses two and three, which I address below. 

Supplementary Table 1 reports IRRs of deaths using negative binomial regression with significant estimates (95 % CIs) in bold. Negative binomial regression is similar to Poisson regression but generates unbiased standard errors in case of overdispersion (Cameron and Trivedi, 2010[2]). In line with earlier arguments, I used week one after vaccination as a reference period, and Supplementary Figure 1B visualizes the data where the vertical axis is log-transformed. 

Concerning the estimate of all doses combined (controlling for each dose, which I address below), the IRR is significantly positive (95 % CI) in 10 of 11 weeks. It implies a significantly increased risk of dying in 10 of 11 weeks compared to week one after vaccination. In weeks four, six, and nine, the IRR is two or higher, implying estimates of at least a double risk of dying compared to week one. The pattern is similar for each dose, e.g., the IRR is 1.95 (95 % CI 1.15-3.39) and 2.58 (95 % CI 1.33-5.03) four weeks after doses one and two, and 2.67 (95 % CI 1.04-6.81) six weeks after dose three. 

Supplementary Table 1 further shows that the IRR after doses two and three is significantly lower compared to dose one (95 % CIs), implying a reduced risk of dying (as noted, Supplementary Figure 1A indicates a similar pattern). The reasons can be several. First, as those never taking COVID-19 vaccines were relatively deprived at the outset (UK Office for National Statistics, 2023[13]), it is not unlikely that those taking only one dose were also relatively deprived compared to those taking two or more. Second, those most severely affected by dose one may either have died or abstained from further doses. Third, as some COVID-19 vaccine batches have induced more side effects than others (Schmeling et al., 2023[12]), it may have induced a relatively high risk of dying after dose one.

Returning to the topic of deaths in the weeks after vaccination, Supplementary Figure 1C graphs an analysis where time is modeled as a continuous variable. Further, it graphs the probability of death after all doses combined as a function of time in weeks since vaccination, modeled as a second- and third-degree polynomial. The analysis applies logistic regression using cluster robust standard errors (Cameron and Trivedi, 2010[2]) regarding week numbers where the time variable is mean-centered to minimize multicollinearity. Also, it controls for each dose (I do not report statistical details, but they show similar results as those above). Supplementary Figure 1C shows that the probability of death increases during the first weeks since vaccination, and Supplementary Figure 1D, reporting marginal effects on the probability (Williams, 2012[14]), shows that the increase is significant in the first four weeks as the low 95 % CIs take above zero values. Following a significant increase, Supplementary Figure 1C shows a flattening curve that then decreases significantly during a few weeks as the high 95 % CIs take below zero values, according to Supplementary Figure 1D. Altogether, the graphs reveal a negative significant second-degree polynomial taking an inverted U-shape. They further indicate a subsequent uptick in the probability of deaths in the out-of-sample estimates for weeks 13 and 14, aligned with a positive third-degree polynomial, but which is non-significant. 

Taken together, among young people in England aged 12-29 years, deaths increased significantly (95 % CIs) in 10 of 11 weeks after COVID-19 vaccination compared to the first week. In three of those weeks, deaths doubled. The pattern was the same for each dose and diverged from Nafilyan et al. (2023[9]), analyzing similar data from England, likely because they compared deaths during the first 12 weeks after vaccination with later deaths. Hence, Nafilyan et al.'s conclusion hinged on assuming a zero increased risk of dying more than 12 weeks after vaccination, but if the risk were increased, it could mask the findings I uncovered.

Also, there was a significant increase in the probability of deaths in the first weeks since vaccination, followed by a significant decrease. Out-of-sample estimates additionally indicated another uptick in weeks 13 and 14, but they should be cautiously interpreted since the trend was non-significant. I nonetheless encourage future research to include more observations beyond the 12 weeks for which data were publicly available. The encouragement is further motivated by Nafilyan et al. (2023[9]), who showed fewer deaths between the first and 24th week after vaccination than later among young people in England (Supplementary Figure 7a in their study). A likely interpretation is that the long-term mortality increased, which disturbingly coincides with other data showing long-term excess mortality in similar age cohorts (Wilthil, 2024[16]). Accordingly, I recommend that future research thoroughly investigates COVID-19 vaccination as a potential explanation for those unfortunate long-term trends.

To conclude, this research letter found that deaths among young people in England increased significantly (95 % CIs) in numerous weeks after COVID-19 vaccination compared to the first week. Based on the findings, I recommend cautiousness concerning further COVID-19 vaccination, particularly among young people, till we know more about underlying causes, e.g., vaccination type or specific batches. 

## Declaration

### Funding

This study received no external funding.

### Conflict of interest

The author declares no conflicting interests. 

### Data availability

All data used are publicly available. 

## Supplementary Material

Supplementary information
