# Supplementary material for: Deaths among young people in England increased significantly in 10 of 11 weeks after COVID-19 vaccination and doubled in three
Source: EXCLI J. 2024 Jul 4;23:908–11. doi: 10.17179/excli2024-7498 (PMC11382299; doi:10.17179/excli2024-7498)
Supplement: Supplementary information [file EXCLI-23-908-s-001.pdf]

EXCLI Journal 2024;23:908-911 – ISSN 1611-2156
Received: June 08, 2024, accepted: July 01, 2024, published: July 04, 2024

Supplementary information to:

Letter to the editor:

DEATHS AMONG YOUNG PEOPLE IN ENGLAND INCREASED SIGNIFICANTLY IN 10 OF 11 WEEKS AFTER COVID-19 VACCINATION AND DOUBLED IN THREE

Jarle Aarstad

HVL Business School, Western Norway University of Applied Science,
PO Box 7030, NO-5020 Bergen, Norway, E-mail: jarle.aarstad@hvl.no

https://dx.doi.org/10.17179/excli2024-7498

This is an Open Access article distributed under the terms of the Creative Commons Attribution License (http://creativecommons.org/licenses/by/4.0/).

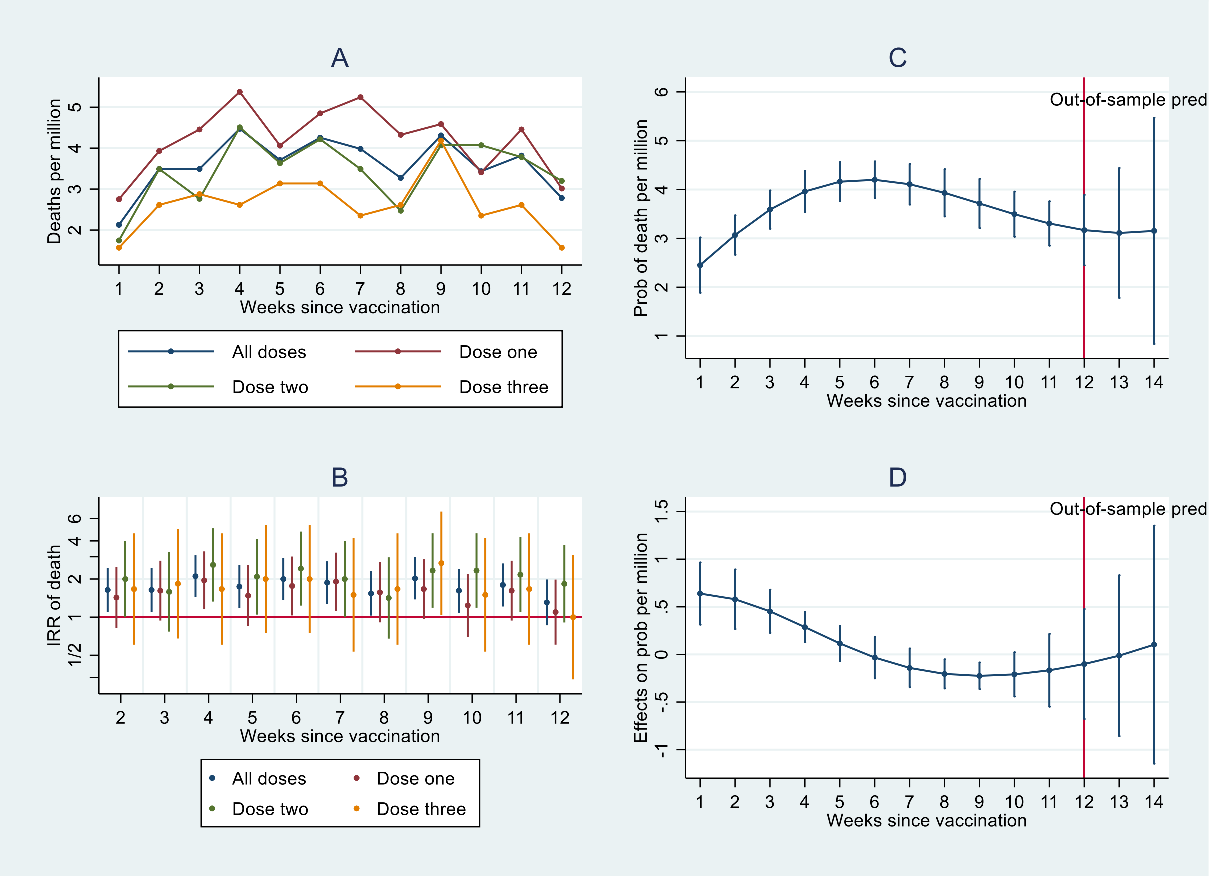

Figure 1: Deaths per million in each week since COVID-19 vaccination (**A**), IRRs of deaths in weeks since COVID-19 vaccination compared to week one with the vertical axis log-transformed (**B**), probability of death per million with weeks since vaccination as a continuous variable (**C**), and marginal effects on the probability of death per million with weeks since vaccination as a continuous variable (**D**). 95 % CIs are reported concerning B, C, and D.

EXCLI Journal 2024;23:908-911 – ISSN 1611-2156
Received: June 08, 2024, accepted: July 01, 2024, published: July 04, 2024

Table 1: IRRs of deaths using negative binomial regression. Significant IRRs (95 % CIs) are in bold.

	All doses			*Dose one*			*Dose two*			*Dose three*		
Week	IRR	95% CIs		IRR	95% CIs		IRR	95% CIs		IRR	95% CIs	
1	Ref			Ref			Ref			Ref		
2	**1.64**	1.10	2.44	1.43	.818	2.50	**2.00**	1.00	4.00	1.67	.606	4.59
3	**1.64**	1.10	2.44	1.62	.940	2.79	1.58	.769	3.26	1.83	.678	4.96
4	**2.10**	1.44	3.08	**1.95**	1.15	3.30	**2.58**	1.33	5.03	1.67	.606	4.59
5	**1.74**	1.18	2.58	1.48	.848	2.57	**2.08**	1.05	4.15	2.00	.751	5.33
6	**2.00**	1.36	2.94	**1.76**	1.03	3.01	**2.42**	1.23	4.74	2.00	.751	5.33
7	**1.87**	1.27	2.76	**1.90**	1.12	3.23	**2.00**	1.00	4.00	1.50	.534	4.21
8	**1.54**	1.03	2.30	1.57	.909	2.72	1.42	.677	2.97	1.67	.606	4.59
9	**2.03**	1.38	2.97	1.67	.970	2.86	**2.33**	1.19	4.59	**2.67**	1.04	6.81
10	**1.62**	1.08	2.41	1.24	.697	2.20	**2.33**	1.19	4.59	1.50	.534	4.21
11	**1.79**	1.21	2.66	1.62	.940	2.79	**2.17**	1.09	4.29	1.67	.606	4.59
12	1.31	.862	1.98	1.10	.606	1.98	1.83	.907	3.70	1.00	.323	3.10
Dose one	Ref											
Dose two	**.821**	.705	.957									
Dose three	**.627**	.511	.769									
